# Evaluation of quality of the children’s menu in mall’s restaurants

**DOI:** 10.1590/1984-0462/2022/40/2021027IN

**Published:** 2022-05-11

**Authors:** Caroline Barboza Duarte, Monica Glória Neumann Spinelli, Andrea Carvalheiro Guerra Matias

**Affiliations:** a Universidade Presbiteriana Mackenzie, São Paulo, SP, Brazil.

**Keywords:** Restaurants, Menu planning, Children, Restaurantes, Planejamento de cardápio, Crianças

## Abstract

**Objective:**

To evaluate the quality of children’s menus in restaurants located in shopping malls.

**Methods:**

To select the sample, restaurants from 30% of shopping malls in each region of the city of São Paulo were included and, after considering only one restaurant per chain, the total was limited to 151 restaurants, 30.2% of which (n=35) presented a children’s menu. Data were collected through a form on Google Forms.

**Results:**

Of the restaurants with children’s menu, 60% (n=21) were conventional restaurants and 40% (n=14) takeaway/fast-food. The large number of chains present in most visited malls showed a democratization of the way of eating, with popular and accessible menus, regardless of social status. Most of the analyzed foods were cooked (41.5%). Most preparations did not use grease in their preparation and there was a notable lack of fruit and vegetables (FV) (4%). Sweet desserts were offered in 11.4% of the places and 20% included gifts with meals.

**Conclusions:**

The scarce offer of children’s menus, few options and low FV offer indicate the need for a new look at the development of children’s menus and a greater integration between the possibilities of restaurants and the expectations of parents and children, in the challenge of integrating the relationship between the supply of new foods that promote healthier habits and their consumption.

## Resumo

**Objetivo:**

Avaliar a qualidade dos menus infantis de restaurantes localizados em shopping centers.

**Métodos:**

Para a seleção da amostra foram incluídos os restaurantes de 30% dos shopping centers de cada região da cidade de São Paulo e, após considerar-se somente um restaurante por rede, o total ficou limitado a 151 restaurantes, dos quais 30,2% (n=35) apresentaram cardápio infantil. Os dados foram coletados por meio de um formulário no Google Forms.

**Resultados:**

Dos restaurantes com menu infantil, 60% (n=21) eram convencionais e 40% (n=14) eram de takeaway/fast-food. O grande número de redes presente na maioria dos shopping centers visitados mostrou a democratização da forma de comer, com cardápios populares e acessíveis, independentemente de classe social. A maior parte dos alimentos analisados era cozida (41,5%). A maioria das preparações não utilizava gordura em seu preparo e foi notável a falta da oferta de frutas, legumes e verduras (FLV) (4%). Sobremesas doces eram oferecidas em 11,4% dos locais e 20% incluíam brindes com as refeições.

**Conclusões:**

A escassa oferta de menus infantis, as poucas opções e a baixa oferta de FLV indicam a necessidade de um novo olhar no desenvolvimento de cardápios infantis e de um maior entrosamento entre as possibilidades dos restaurantes e as expectativas de pais e crianças, no desafio de integrar a relação entre a oferta de novos alimentos, que promovam hábitos mais saudáveis, e o seu consumo.

## INTRODUCTION

According to the Brazilian Society of Pediatrics,^1^ healthy eating requires sufficient nutrient intake for children to achieve normal growth and development, as well as the prevention of food-related illnesses.

Many of these illnesses reflect the changes in lifestyle presented in new data from the Brazilian Institute of Geography and Statistics (*Instituto Brasileiro de Geografia e Estatística* – IBGE) (2017–2018), which show that 32.8% of the Brazilian population has expenses with food outside the home, and that higher percentages of eating outside the home occurred in the Midwest (38.0%) and Southeast (34.2%) regions, above the national average.^2^

This new food practice of the Brazilian population, which has been changing in recent decades mainly due to changes in daily life and work, is the result of multiple factors that mark contemporaneity, such as: urbanization, growing industrialization and multiplicity of the attributions of women, who for a long time were responsible for feeding the family. These factors contribute to reinforce the consumption of food outside the home, as well as the pursuit for practicality and saving time.^3,4^

Shopping malls are the third most frequented place for North Americans, preceded by home and work,^5^ while Brazilians tend to visit the mall at least twice a month.^6^ The study by Pereira Filho et al.^7^ showed that Brazilians see this format of shopping center as a privileged place for shopping and leisure. The variety and diversity of restaurants in food courts prove to be extremely attractive for those who eat outside the home. These establishments, which serve members of the urban middle class, have become flexible, practical, and accessible spaces for meals at moderate cost, both in moments of leisure and during meal breaks that occur during the work or study day, this cost being one of the most sought-after factors in shopping malls.^6^

Restaurants are a regular-use environment for many families. It is estimated that every day, in the United States of America, one third of school-age children visit fast-food restaurants. Previous analysis of the menus offered to most children (*i.e.*, the meals listed on children’s menus) showed that they were of poor quality based on criteria such as insufficient amounts of vitamins A and C, calcium, iron, fiber, fruit, vegetables or whole grains, and that children’s consumption of restaurant food has been associated with higher daily intakes of calories, saturated fat, sodium, and sugar.^8^

Considering the increase in this habit by families and the reports of poor nutritional quality of menus currently offered in restaurants to children, and assuming that changing these foods has the potential to improve the quality of the diet, alleviate excessive energy consumption, and help shape healthy habits, this study aimed to assess children’s menus in shopping mall restaurants in the city of São Paulo.

## METHOD

The present study is cross-sectional, with quantitative and qualitative analyses carried out between 2019 and 2020. For sample selection, there was a total of 48 shopping malls registered in the city of São Paulo in the researched period, and approximately 30% of which were selected according to each region, a value established by convenience, through drawing lots. All restaurants in each selected shopping mall were surveyed and only those serving a children’s menu were included in the study. For restaurants of the same chain established in more than one shopping mall, with identical menus, only one of them was evaluated. Exclusion criteria were restaurants that did not have a children’s menu, those with a self-service buffet by weight, kiosks or islands.

Data collection was carried out online using Google Forms. The questionnaire, in digital format (Google Forms), was filled out in an online live session, in duplicate, and under observation by the researchers who collected data on the menus directly at the sites. Each form was filled out by two independent evaluators, aiming to minimize errors.

The menus were qualitatively evaluated based on the recommendations of the Food Guide for the Brazilian Population^9^ and the Brazilian Society of Pediatrics^10^ and the Qualitative Assessment of Menu Patterns method (*Avaliação Qualitativa de Padrões de Cardápio* – AQPC method).^11^ The AQPC method uses the following criteria: cooking techniques used in cooking preparations, offering fried foods in isolation and also associated with sweets or fatty meats, which allows the assessment of the risk involved in the association of excess lipids with simple carbohydrates. The method also assesses the supply of important items for a nutritionally adequate diet, such as fruits and vegetables (FV), disregarding small portions of vegetables such as those present in hamburgers.^11^

Data collection approached the characteristics and identification of the catering establishment, the type of establishment, whether there is a children’s menu, the options on this menu, the listing of its component items, information on allergens, the nutritional information statements, prices, and gifts or attractions for children.

The results were presented in the form of descriptive statistics to analyze the differences between the characteristics of children’s menus according to the type of establishment (takeaway/fast-food *versus* restaurants). The chi-square test was used for qualitative variables and the Student’s *t*-test for quantitative variables. Statistical analyses were performed using the Statistical Package for Social Sciences (SPSS), version 21, considering a significance level of 5% for all statistical tests.

Complementarily, it is important to communicate that this work is part of a larger project, entitled “Quality in Food Services” (“*Qualidade em Serviços de Alimentação*”), and is being developed together with other countries in order to assess and discuss the quality of children’s menus. Data collection took place in an observational manner, without any direct or indirect personal contact with humans.

## RESULTS

It must be considered that, as the scope of the study was to assess the menus available to children, it was decided to maintain a single restaurant to represent a network, since the menu is the same, although it is not possible to ignore that these networks, as they are present in most shopping malls, reach a considerable audience.

A total of 344 establishments were listed, of which only 93 (37.1%) provided a children’s menu and 251 (72.9%) did not. After the selection of restaurants with a children’s menu, only one establishment was considered to represent each chain. As a result, the final total of restaurants with a children’s menu was limited to 35 units. Of the establishments with a children’s menu, 40% were classified as takeaway/fast-food, of quick service, no waiting and with orders placed at the counter,^12^ and the other 60% as conventional restaurants, with orders placed at the table (Table 1).

**Table 1.** Distribution of locations according to the presence of a children’s menu. São Paulo, 2019–2020.

	With a children’s menu		Without a children’s menu		Total	
	n	%	n	%	n	%
Total of establishments visited	93	37.1	251	72.9	344	100
Considering 1 establishment per chain	35	30.2	116	76.8	151	100

The average price found for adult menus was R$ 49.51, and the average price for children’s menu was R$ 26.55, corresponding to practically half the value of the first one, which shows the importance of valuing the quality of this menu, since its value can induce the purchase by the economic advantage for the parents, considering that children eat less.

Some places had different combination possibilities for the children’s menu, but most, or 54% (n=19), offered only one to three options, 26% (n=9) offered four to ten options, and the remaining 20% (n=7) more than 11 combinations. The three most common types of preparation were boiled, grilled, and fried, respectively (Table 2).

**Table 2.** Distribution in percentage of preparations according to the cooking method (n=202). São Paulo, 2019–2020.

Cooking method	n	%
Cooked	84	41.5
Fry	41	20.3
Grill	57	28.2
Uncooked — raw	8	4.0
*Sauté*	3	1.5
Steam	3	1.5
Total	202	100

Several characteristics of the children’s menu of the establishments were analyzed, evaluated according to the type of establishment, and those with a statistically significant difference are shown in Table 3.

**Table 3.** Distribution of characteristics of establishments according to type (conventional versus takeaway/fast-food). São Paulo, 2019–2020.

Characteristic	Conventional		Takeaway/fast-food		Total		p-value
	n	%	n	%	n	%	
Pasta							
No	4	19.1	9	64.3	13	37.1	0.007
Yes	17	80.9	5	35.7	22	62.9	
Buttered pasta							
No	11	52.4	13	92.9	24	68.6	0.012
Yes	10	47.6	1	7.1	11	31.4	
Total	21	100	14	100	35	100	

In takeaway/fast-food establishments, statistically more photos/illustration of the menu (p=0.019) were observed than in conventional restaurants.

When analyzing the differences between a chain restaurant and a restaurant without a chain, there was also no statistically significant difference — 75% (n=12) of those not belonging to a chain and 64.52% (n=120) of those with a chain did not use grease in cooking.

Regarding FV, there were no statistically significant differences regarding the type of restaurant (Table 4). The same did not occur when examining the question of being a chain restaurant or not (p=0.034): there was no FV in 81.25% of the non-chain restaurants and in 53.76% of the chain restaurants.

**Table 4.** Distribution of establishments according to type (takeaway/fast-food or conventional) and characteristics of preparations that make up the children’s menu (type of cooking and presence of fruits and vegetables). São Paulo, 2019–2020.

Characteristics		Type of establishment				Total		p-value
		Takeaway/fast-food		Conventional				
		n	%	n	%	n	%	
Cooking	Without grease	43	65.15	89	65.44	132	65.35	0.968
	With grease	23	34.85	47	34.56	70	34.65	
FV	Absent	40	60.61	73	53.68	113	55.94	0.352
	Present	26	39.39	63	46.32	89	44.06
Total		66	100	136	100	202	100	

FV: fruits and vegetables.

As for desserts, of the 35 children’s menus analyzed, 11.4% (n=4) had sweets such as milkshakes and ice cream, preparations with high sugar content and usually made with grease, often accompanied by dishes with fried and/or greasy foods.

As for the offer of collectible gifts associated with the children’s menu, this was a situation found in 20% of the restaurants as a way of attracting children’s attention.

## DISCUSSION

The large number of chains identified in the shopping malls visited in this study (Table 1) showed that they are present in all types of shopping malls, regardless of their category and/or location, which shows that there is currently a democratization of food that is not exclusive of social, economic, or educational level.

Regarding Table 1, a result similar to that obtained by DuBreck et al.^13^ was obtained, showing that children in areas of low social and economic level would have a menu very similar to that of a child living in an area of higher economic status, with respect to out-of-home consumption. They also observed low quality menus with the same items, such as fried chicken, pizza, cheeseburgers, and so on.

Offering menus adapted for children should represent a possibility of better nutrition for this group, when eating away from home. Only 37.1% of the visited establishments had a kids’ menu, considerably reducing the sample size to be surveyed. Hill et al.^14^ found in the United States (Virginia and North Carolina) the offer of children’s menus in less than half (46%) of the restaurants in that region. Most of the menus for children (Table 1) were found in conventional restaurants (60%), a result similar to the study by Serrano and Jedda,^15^ which showed 69.7% of the presence of these menus in conventional restaurants.

A comparative study of the prices of children’s menus between Portugal and Brazil showed similarity between the two countries; however, as the Portuguese minimum wage is higher, that country possibly allows greater access to these menus, almost always of lower nutritional quality.^16^

Most children’s menus (54%) did not have great combination possibilities. This fact was also verified by Anzman-Frasca et al.,^8^ as restaurants in general described barriers regarding the modification of children’s menus, which are more static than adult menus. There was unanimity in saying that children’s menus are pretty fixed, with new items integrated about once a year, and they are not a key profit factor, so there is little incentive for restaurants to invest in modifications.

With regard to the various cooking methods used in food offered in restaurants, only 1.5% of the food, such as vegetables, was steamed; 1.5% was *sautéed*, usually in butter, and 8% was offered fresh (Table 2). Of the 202 preparations, a fifth was offered in fried form. Abroad, according to DuBreck et al.,^13^ just under a third (31%) of the restaurants offered an unfried vegetable dish, such as salad or steamed broccoli. For Hill et al.^14^ the values were even lower: 12 (9%) offered a secondary item of unfried vegetables. According to Lee et al.,^17^ steamed vegetables retain a higher concentration of vitamin C than when boiled, due to the reduced contact with water and the relative low temperature. The use of minimal amounts of water and cooking for short periods result in greater retention of vitamins.

Pasta was the dish with the greatest statistical significance (Table 3), and conventional restaurants were the ones that offered more of this type of preparation compared to takeaway/fast-food. The results show that food monotony is often present in restaurants that offer pasta on the children’s menu — they only offer pasta with different types of sauce and rarely accompanied by one type of meat or salad. In addition to pasta, food monotony was also represented by the form of preparation, such as frying, found twice or more in the same dish in 40% of selected menus, thus constituting dishes with a high content of saturated fat. Food monotony, without variations in the type of food and preparations, is a factor that can contribute to reduced appetite and the child’s lack of interest in consuming food. Therefore, a balanced diet should be composed of colorful meals, with different textures and shapes, making the dish attractive for children.^10^

The strategy of using illustrations in children’s menus is mainly used in takeaway/fast-food restaurants, as studies claim that brand identity can influence young children’s perceptions of taste. Images can be used as a fun and creative way to catch children’s attention, such as food in fun and colorful formats.^18,19^

A study by Rehm and Drewnowski^20^ showed that, since 2004, fast-food restaurants are contributing less to the percentages of fat in the diets of children between four and 11 years old. In 2003–2004, fast-food restaurants contributed with 17.4% of total fat, a statistically higher value than the 13.8% in 2009–2010. However, a study carried out in Chicago by Powell and Nguyen^21^ showed that conventional restaurants and especially fast-food restaurants are associated with higher consumption of total and saturated fats and excess calories. The same was observed in a study carried out by Duarte,^12^ which showed that 71% of the restaurants reported had values above the expected level of total fat. Excessive consumption of saturated fats (present in oils and fats) increases the risk of heart disease, obesity, and several other chronic diseases, in addition to providing a high amount of calories per gram.^9,22^

With regard to FV, compared to those by Viegas et al.,^16^ the results of the present study are more favorable, although they were not evaluated quantitatively, with only the presence or absence of the food group being observed. According to Anzman-Frasca et al.,^8^ restaurant owners reported difficulties in implementing healthy foods in children’s menus due to the barriers of eating habits that are little exposed to different and healthy foods. Hill et al.^14^ observed that 39% of the restaurants offered fruit, although in only 23.4% of them they were offered without added sugar. Rehm and Drewnowski^20^ reported a slight but significant increase in fruit consumption among fast-food restaurants since 2004, thanks mainly to increased consumption of whole fruit. Other studies, such as those by Horta, Junior and Santos^23^ and Leal et al.,^24^ found that the diets of Brazilian children are of poor quality, with low FV intake. This intake must be encouraged, as these products are the basis of a nutritionally balanced, tasty, culturally appropriate diet that promotes a socially and environmentally sustainable food system. The increase in consumption can result in a reduction in the intake of food products with high energy density and low nutritional value, such as those with added sugar and fat, common in processed foods and fast-foods.^9,22^

The results showed that few restaurants offered sweet desserts — a positive factor, since, according to the Brazilian Society of Pediatrics,^10^ foods rich in fats and sugars, such as ice cream, lead to nutritional disorders from childhood to adulthood, such as chronic non-communicable diseases. Foods with sugar are preferred by children, as the sweet taste is innate to human beings; it is up to parents to guide what to consume and control the daily amount of sugar.

Also in the DuBreck et al.^13^ study, a large part of the menus (43.6%) included dessert in children’s meals, but only 4% offered healthy desserts, such as fresh fruit. Similarly, in the research by Hill et al.,^15^ it was observed that only ten (7.3%) of the 137 establishments included a dessert as part of the children’s meal; however, none offered healthy desserts.

As for the offer of gifts with meals, the results suggest that, in the case of children, the criteria for choosing food and toys are possibly influenced by television advertisements and that family purchases are determined by these requests. The present study found that 20% of the surveyed restaurants offered freebies with meals. In the survey by Hill et al.,^14^ 29% of the establishments included a toy in the children’s meal and 8% used brand marketing to promote this meal. Both the Food Guide for the Brazilian Population^9^ and the Food Guide for Brazilian Children under 2 years of age^24^ recommend that, for a healthy diet, one should eat regularly and carefully, and it is not advisable for children to be fed in environments with many attractions, at risk of generating disinterest in food. There is a consensus that, when the meal is received using distractions, the feeding occurs automatically, without paying attention to the food, which can lead to future damage such as loss of control of the hunger and satiety mechanism, in addition to excessive gain or weight loss.^25^ This matter was a limitation of the study, which evaluated the quality, but not the quantity, of foods imposed on children’s menus, both from the point of view of portion sizes and consumption. Only the offer of preparations was considered, which limits knowledge of the preferences of both parents and children when choosing food.

Given the aforementioned facts, it is necessary to highlight the importance of food consumption outside the home, despite the fact that there is a notorious shortage of children’s menus in restaurants. The few possibilities for combinations of dishes, the limited supply of FV and the monotony of food indicate the need for a new look in the development of children’s menus and a greater relationship between the possibilities of implementing restaurants and the expectations of parents and children, encouraging new healthier foods and/or forms of preparation. Furthermore, it is necessary to work with those responsible for the children, as the problem is not only in the offer of children’s menus, but also in the food choice made by customers. It is also pertinent to make nutritional information available to consumers in a simple, direct, and clear way.
